# Tumor Antigenicity and a Pre-Existing Adaptive Immune Response in Advanced BRAF Mutant Colorectal Cancers

**DOI:** 10.3390/cancers14163951

**Published:** 2022-08-16

**Authors:** Elena Bolzacchini, Laura Libera, Sarah E. Church, Nora Sahnane, Raffaella Bombelli, Nunzio Digiacomo, Monica Giordano, Guido Petracco, Fausto Sessa, Carlo Capella, Daniela Furlan

**Affiliations:** 1 Oncology Department, Sant’Anna Hospital, ASST-Lariana, 22100 Como, Italy; 2 Unit of Pathology, Department of Medicine and Surgery and Research Center for the Study of Hereditary and Familial Tumors, University of Insubria, 21100 Varese, Italy; 3 NanoString Technologies Inc., Seattle, WA 98109, USA; 4 ASST Sette-Laghi, 21100 Varese, Italy; 5 Oncologia Medica Humanitas Gavazzeni, 24125 Bergamo, Italy; 6 Pathology Department, Ospedale S. Anna, ASST Lariana, 22100 Como, Italy

**Keywords:** BRAF mutation, colorectal cancer, immune checkpoint inhibitors, PanCancer IO 360, NanoString, MSI, CD8+, MHC Class I antigen

## Abstract

**Simple Summary:**

BRAF mutant metastatic CRCs (BRAF-mCRCs) are considered a unique clinical entity characterized by a dismal prognosis and that do not respond efficiently to both standard chemotherapy and to orally selective inhibitors of BRAF^V600E^. In this study, the gene expression profiles of 89 immunotherapy-naïve BRAF-CRCs were generated using the PanCancer IO 360 gene expression panel to improve the knowledge of the mechanisms involved in tumor-suppressive immune functions in BRAF-mCRCs. A significant fraction of BRAF-mCRCs shows a hot/inflamed profile and may be potential candidates for responding to immunotherapy. Only a partial overlap between these hot signatures and the presence of microsatellite instability (MSI) was observed, demonstrating that MSI tumors showed a not differential expression of MHC Class I antigen presentation pathway compared with microsatellite-stable tumors. The analysis of gene expression profiles is a promising strategy both for immune profiling of primary tumors before any treatment and for following the evolution of metastatic disease during therapy.

**Abstract:**

The main hypothesis of this study is that gene expression profiles (GEPs) integrating both tumor antigenicity and a pre-existing adaptive immune response can be used to generate distinct immune-related signatures of BRAF mutant colorectal cancers (BRAF-CRCs) to identify actionable biomarkers predicting response to immunotherapy. GEPs of 89 immunotherapy-naïve BRAF-CRCs were generated using the Pan-Cancer IO 360 gene expression panel and the NanoString nCounter platform and were correlated with microsatellite instability (MSI) status and with CD8+ tumor-infiltrating lymphocyte (TIL) content. Hot/inflamed profiles were found in 52% of all cases, and high scores of Tumor Inflammation Signature were observed in 42% of the metastatic BRAF-CRCs. A subset of MSI tumors showed a cold profile. Antigen Processing Machinery (APM) signature was not differentially expressed in MSI tumors compared with MSS cases. By contrast, the APM signature was significantly upregulated in CD8+ BRAF-CRCs versus CD8− tumors. Our study demonstrates that a significant fraction of BRAF-CRCs may be a candidate for immunotherapy and that the simultaneous analysis of MSI status and CD8+ TIL content increases accuracy in identifying patients who can potentially benefit from immune checkpoint inhibitors. GEPs may be very useful in expanding the spectrum of patients with BRAF-CRCs who can benefit from immune checkpoint blockade.

## 1. Introduction

*BRAF* is considered a driver gene of MLH1 methylation in sporadic colorectal cancer (CRC) [1]. Globally, *BRAF* V600E mutation is found in about 40–60% of MSI CRC and only in 5% to 10% of microsatellite-stable (MSS) CRC [2].

Clinically, the *BRAF* mutation is an enigmatic target in CRC, and regardless of MSI status, BRAF mutant metastatic CRCs (mCRCs) are considered a unique entity characterized by dismal prognosis (median overall survival of fewer than 12 months) that do not respond efficiently to both standard chemotherapy and to orally selective inhibitors of *BRAF*^V600E^ [3].

In the randomized phase 3 KEYNOTE-177 trial, pembrolizumab reduced the risk of disease progression or death by 40% compared with standard treatment when received as first-line therapy for MSI or mismatch repair deficient (dMMR) metastatic CRC [4]. Previous studies of MSI-H–dMMR tumors showed higher complete response rates with pembrolizumab or other immune checkpoint inhibitors than with chemotherapy [5,6,7,8,9,10]; however, 30% of patients treated with pembrolizumab in KEYNOTE-177 had primary resistance.

There is increasing evidence that the clinical benefit from checkpoint inhibitors has been associated both with tumor antigenicity, which is routinely evaluated using MSI/MMR protein expression, and with the presence of a pre-existing adaptive immune response, typically measured by immunohistochemistry to identify a tumor “inflamed” phenotype [11]. Recent studies in early-stage CRC, melanoma, lung cancer, and bladder cancer have shown remarkable and deep pathological responses to neoadjuvant immune checkpoint inhibitors (ICIs) [12,13,14,15] due to a difference in T cell infiltration (TCI), a lower degree of systemic immune suppression, the absence of visceral metastases and a lower tumor burden. Interestingly, Chalabi M et al. [5] showed that neoadjuvant immunotherapy leads to pathological responses in MMR-proficient and MMR-deficient early-stage CRCs, suggesting that CD8+PD-1+Tcell infiltration was the sole biomarker found to predict response in MMR-proficient CRCs. No data are available regarding the potential efficacy of immunotherapy for BRAF-mutant MSS CRCs.

In this scenario, BRAF mutant CRCs (BRAF-CRCs) represent a heterogeneous tumor entity with a high frequency of MSI and/or CD8+ tumors [16,17] that urgently deserve to be investigated to predict both tumor antigenicity and the level of tumor inflammation for potential eligibility for immunotherapy treatments.

The working hypothesis of this study is that the gene expression profiles (GEPs), integrating both tumor antigenicity and a pre-existing adaptive immune response, may be a successful strategy to generate distinct immune-related signatures useful for the assessment of new predictive diagnostic tools for immunotherapy treatments, in line with recent studies [18,19].

The main objectives of this study were to (i) generate GEP of 89 immunotherapy-naïve BRAF-CRCs, (87% of which were advanced CRCs) using the PanCancer IO 360 gene expression panel and the NanoString nCounter platform; (ii) to evaluate the association between MMR status/CD8+ TIL infiltrate and immune-related gene signatures of BRAF-CRCs; (iii) to explore the distribution of Tumor Inflammation Signature (TIS) scores within BRAF-CRCs; (iv) to assess the TIS score’s prognostic value.

## 2. Materials and Methods

### 2.1. Patients and Clinical-Pathologic Study

The study cohort was a retrospective and multicentric series of 89 BRAF-CRCs from 88 patients, encompassing 53 BRAF-mCRCs previously described [17] and 36 new BRAF-CRCs selected from routine diagnostics from January 2018 to December 2019.

Detailed clinical-pathological features (sex, age, primary tumor site, histologic type, stage at the diagnosis, presence of metastasis, grade, growth pattern, tumor budding, necrosis, vascular space invasion, perineural invasion, percentage of tumor stroma, and intratumoral lymphocyte count) and molecular data (BRAF mutation, Microsatellite instability status) are reported for each tumor in Table S1. Moreover, PD-L1, p53, Ki-67, synaptophysin immunohistochemical expression, as well as the type of intratumoral lymphocytic infiltrate (TIL) using anti-CD3 and anti-CD8 antibodies were evaluated. The antibodies’ list and protocols of immunohistochemical expression are reported in Table S2. Clinical data regarding oncological treatment were also collected (chemotherapy regimens and lines of treatment).

The study was performed in accordance with the Declaration of Helsinki, and the ethics committee of Ospedale di Circolo di Varese approved the present study (approval no. 0008465).

### 2.2. RNA Extraction and Hybridization to nCounter Codeset

The RNA was obtained from three representative formalin-fixed and paraffin-embedded sections (FFPE, 8 μm). The RNA was extracted after manual microdissection from 89 FFPE tumor samples and 8 normal colorectal tissue samples from non-neoplastic patients using the Maxwell^®^ RNA FFPE Kit and Maxwell 16 system (Promega, Madison, WI, USA) according to the recommendations of the manufacturer. The RNA was quantified using a Qubit™ RNA XR Assay Kit (Invitrogen–Thermo Fisher Scientific, Whaltam, MA, USA).

Gene expression analysis was conducted on the NanoString^®^ nCounter^®^ gene expression platform (NanoString Technologies, Seattle, WA, USA) using the NanoString PanCancer IO 360^TM^ Panel, which contained 750 genes that cover the key pathways at the interface of the tumor, tumor microenvironment, and immune response. Briefly, per sample, 100–300 ng (67 samples) or 12–99 ng (22 samples) of the total RNA in a final volume of 5 μL was mixed with a 3′ biotinylated capture probe and a 5′ reporter probe tagged with a fluorescent barcode from NanoString PanCancer IO 360 Panel code set. The probes and target transcripts were hybridized overnight at 65 °C for 12–16 h, according to the manufacturer’s recommendations. The hybridized samples were run on the NanoString nCounter preparation station using the high-sensitivity protocol. The samples were scanned at high scan resolution (280 FOVs, fields of view) on the nCounter Digital Analyzer.

### 2.3. Statistical Analysis of NanoString PanCancer IO 360 Panel Data

The raw data for each sample and gene were normalized to internal controls to eliminate technical variability of the assay, and then the counts were normalized to the geometric mean of 20 endogenous housekeeping genes or 10 genes for the Tumor Inflammation Signature (TIS), followed by log2 transformation. Gene expression signatures were calculated as a weighted linear average of the constituent genes [20,21,22]. The weighted scores used for the calculation of the signatures are NanoString intellectual property. Normalized gene counts and signature scores were compared to molecular and immunostaining features. The log2 fold change, Wald-type confidence interval, and *p*-value were calculated for each gene and signature. For the analysis of the survival time, the genes and scores were dichotomized into high and low groups based on median value, except for TIS, which was divided into tertiles.

### 2.4. Statistical Analysis of Clinico-Pathological Data

Statistical analysis was performed using Student’s *t*-test and ANOVA followed by the Bonferroni test and Pearson chi2-test. Survival curves were calculated using the Kaplan–Meier estimator test. A *p*-value of <0.05 was considered significant. The Stata Statistical Software release 17 (College Station, TX, USA: StataCorp LLC) was used for the statistical analyses.

## 3. Results

### 3.1. MMR Status, CD8+ TIL Infiltrate and Clinical-Pathological Features of BRAF-CRCs

Metastatic disease (stage IV according to TMN) was the initial diagnosis for 50% of the patients, and 36% of the patients had developed a relapse after a diagnosis of early-stage CRC (stage I–III). Only 12 patients (14%) did not show evidence of metastatic disease in a mean follow-up time after surgery of 62 months.

As expected, BRAF-CRCs showed a high frequency of MSI tumors (48% of cases). These tumors were MLH1 deficient and were characterized by the well-known clinical-pathological features associated with MSI, such as older age onset, right tumor site, specific histological types (mucinous or medullary/undifferentiated tumors), high histological grade, and high content of CD8+ TILs.

Interestingly, when we evaluated the relationship between MMR status and CD8+ TIL content, we found only a partial overlap between the two subgroups (Table 1). Indeed, although a highly significant association was observed between MSI and CD8+ TIL expression (27 MSI CRCs were CD8+ and 34 MSS cases showed CD8 negativity, *p* = 0.0001), 15 out of 43 MSI tumors (35%) were CD8- (MSI/CD8-) and 12 out of 46 MSS tumors (26%) were CD8+. These findings confirm our preliminary results in a smaller series of BRAF-mCRCs [17], suggesting that a pronounced host immune reaction is not unique to MSI BRAF-mCRC and that simultaneous evaluation of MSI status and CD8 T-cell content could be a useful strategy for identifying a prognostically distinct subgroup of patients with potential eligibility for cancer immunotherapy drugs.

Considering the 76 metastatic patients, 50 (66%) underwent first-line therapy for metastatic disease with chemotherapy (doublet or triplet) +/− targeted therapy (anti-vascular endothelial growth factor [VEGF] or anti-epidermal growth factor receptor [EGFR] antibody), 18 patients (24%) received a second-line therapy (doublet or mono-chemotherapy +/− targeted therapy) and only 10 patients (13%) received a third line therapy (regorafenib or TAS 102). Palliative care was proposed for 34% of the patients at the diagnosis of metastatic disease.

### 3.2. High and Low Immune Profiles of BRAF-CRCs

Using nCounter gene expression data, we evaluated the GEPs of all 89 BRAF-CRCs.

The PanCancer IO 360™ Panel includes 750 genes involved in the interactions between the immune system and cancer cells, generating a total of 48 signatures (Table 2) that measure: (1) tumor immunogenicity and tumor escape from immune surveillance (orange color); (2) microenvironment including stromal factors and inhibitory metabolism pathways (green color); (3) immune response including inhibitory immune signaling, anti-tumor immune activity, and immune cell abundance signatures (blue color).

**Figure 1:**
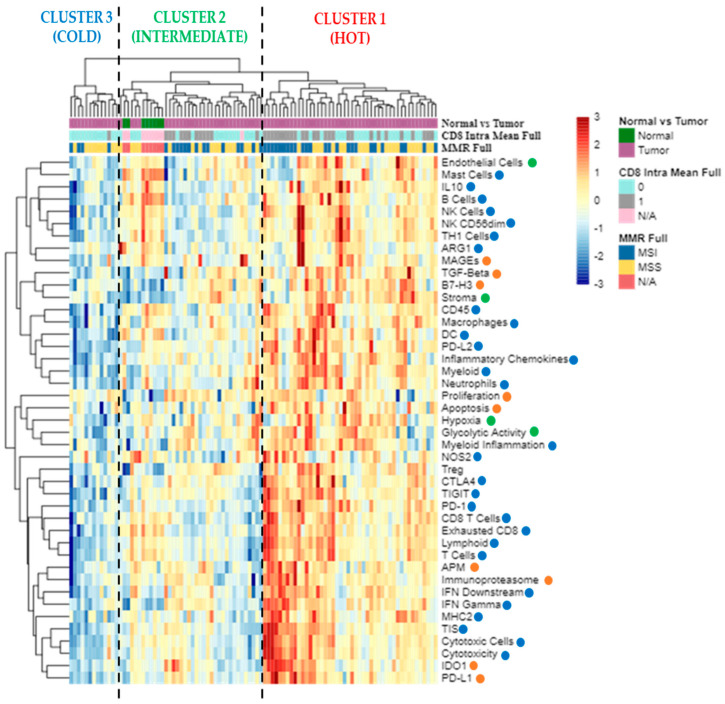
The ‘All Signatures’ heatmap uses unsupervised hierarchical clustering to show relatedness among signature scores for each sample. Scores are scaled by signature to have a mean of zero and a standard deviation of one. The standardized signature scores are truncated at ±3 standard deviations to preserve greater clarity in color change within the largest proportion of data (99% of the data should fall within ±3 standard deviations of the mean). Sample annotations are listed at the top of the heatmap: tumor vs. normal samples (violet vs. green); CD8 positive (category 1) vs. CD8 negative (category 0) CRCs (grey vs. light blue); MSI vs. MSS tumors (dark blue vs. yellow). The signatures are displayed in rows and listed to the right of the heatmap: red dots correspond to tumor signatures, green dots correspond to microenvironment signatures, and blue dots correspond to immune response signatures. Each column is a unique sample. Orange dots: tumor signatures; green dots: tumor microenvironment signatures; blue dots: immune response signatures; N/A, not available data; MMR, mismatch repair status; MSI, microsatellite unstable; MSS, microsatellite stable.

Considering all signatures, the unsupervised hierarchical clustering analysis identified three major clusters (Figure 1): cluster 1 is characterized by a “hot/inflamed” profile (46 BRAF-CRCs, 51.7%), cluster 2 with an intermediate profile (30 BRAF-CRCs, 33.7%, and eight normal mucosae), and cluster 3 showing a cold profile with the downregulation of almost all immune signatures (13 BRAF-CRCs, 14.6%). Interestingly, 26 out of 39 CD8+ BRAF-CRCs (66.7%) were enlisted in cluster 1, while 12 CD8+ cases belonged to the intermediate cluster 2 (30.7%) and only 1 CD8+ CRC were found among cluster 3 (cold, 2.6%) cases. Regarding MSI status (MSI in dark blue vs. MSS in yellow, Figure 1), the 43 MSI BRAF-CRCs were distributed as follows: 25 cases in cluster 1 (58.1%), 14 CRCs in cluster 2 (32.6%), and four tumors in cluster 3 (9.3%).

### 3.3. Differential Gene Expression in MSI vs. MSS and in CD8+ vs. CD8− BRAF-CRCs

**Figure 2:**
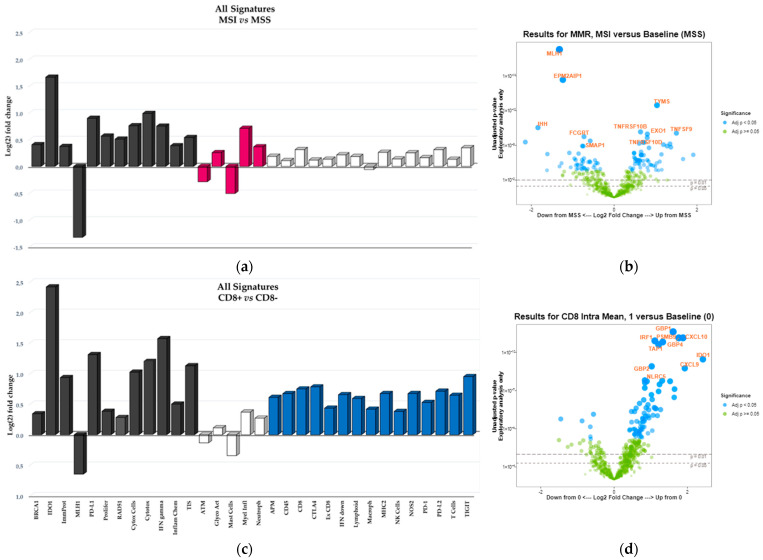
‘All Signatures’ histograms display the 32 signatures that are overexpressed/downregulated in MSI versus MSS samples (**a**) and in CD8+ versus Cd8− samples (**c**). Differential expression versus the baseline group (MSS or CD8-, respectively) is reported on y-axis as signature’s Log(2) fold change. Black bars indicate those signatures that are significantly overexpressed/downregulated in both MSI and CD8+ groups; burgundy bars represent the signatures overexpressed/downregulated in just MSI group, blue bars indicate the signatures overexpressed/downregulated in just CD8+ group; white bars represent the signatures that are not differential expressed versus the baseline groups (*p* > 0.05). Volcano plot displays each gene’s fold change (or difference on the Log(2) scale) and significance (*p*-value) between MSI and MSS samples (**b**) and between CD8 positive (CD8+, category 1) and CD8 negative (CD8−, category 0) samples (**d**), represented along the x-axis, with the significance (*p*-value) along the y-axis. Genes that have greater statistical significance will produce points that are both larger and darker in hue, in addition to appearing higher on the plot. Genes that have greater differential expression versus the baseline group (MSS or CD8−, respectively) appear further from the center of the plot. Genes further to the right indicate an increase in expression, and signatures or genes further to the left indicate a decrease in expression relative to the baseline group. Horizontal lines indicate 0.01 and 0.05 *p*-values; blue dots indicate adjusted *p*-values lower than 0.05; green dots indicate adjusted *p*-values higher than 0.05.

Differential expression analysis between MSI and MSS BRAF-CRCs was performed, allowing the identification of 268 differentially expressed genes belonging to 17 signatures (Figure 2a,b, Table S3). As expected, the top downregulated genes in MSI tumors were *MLH1* and *EPM2AIP1* genes, accordingly to the fact that all MSI BRAF-CRCs collected in this study were characterized by MLH1 protein loss due to gene methylation and that *MLH1* and *EPM2AIP1* share a common promoter whose silencing has been shown to affect both genes. Additionally, MSI cancers showed a significant downregulation of both ATM checkpoint signaling and mast cell abundance, which is a signature measuring macrophages in the tumor microenvironment.

On the contrary, 14 signatures were upregulated in MSI tumors (Figure 2a) and included key factors involved in (i) releasing of cancer antigens and cancer antigen presentation (i.e., *BRCA1*, *RAD51*, key components of immunoproteasome), (ii) inhibitory tumor mechanisms and tumor sensitivity to immune attack (IDO1, PD-L1, genes involved in tumor proliferation), (iii) inhibitory metabolism (genes associated with hypoxia), (iv) immune response signatures namely, TIS that measures the abundance of a peripherally suppressed adaptive immune response within the tumor; Interferon gamma (IFN) signaling and inflammatory chemokines; cytotoxic cells and molecules used by natural killer and CD8+T cells to mount a cytolytic attack on tumor cells (cytotoxicity signature).

Considering CD8-positive T cells, a differential expression between CD8+ and CD8- BRAF-CRCs was observed for 286 genes belonging to 27 signatures (Figure 2c,d, Table S4). Interestingly, 12 out of 27 signatures were the same upregulated/downregulated in both CD8+ and MSI+ BRAF-CRCs (black bars in Figure 2a,c). By contrast, 15 signatures were overexpressed in only CD8+ BRAF-CRCs (blue bars in Figure 2c) and included: (i) immune response signatures measuring lymphoid, macrophage, NK, CD45, and CD8 T cells abundance (ii) Antigen Processing Machinery (APM) that measures the abundance of genes in the MHC Class I antigen presentation pathway and some key genes involved in processing the antigens prior to presentation (iii) Major histocompatibility complex class II antigen presentation; (iv) interferon signaling response and Nitric Oxide Synthase 2 gene expression; (v) exhausted CD8 cell abundance; (vi) inhibitory tumor mechanisms that suppress anti-tumor immune activity including upregulation of PD-L2, PD-1, TIGIT, CTLA4. On the contrary, burgundy bars in Figure 2a indicated MSI-specific signature.

**Figure 3:**
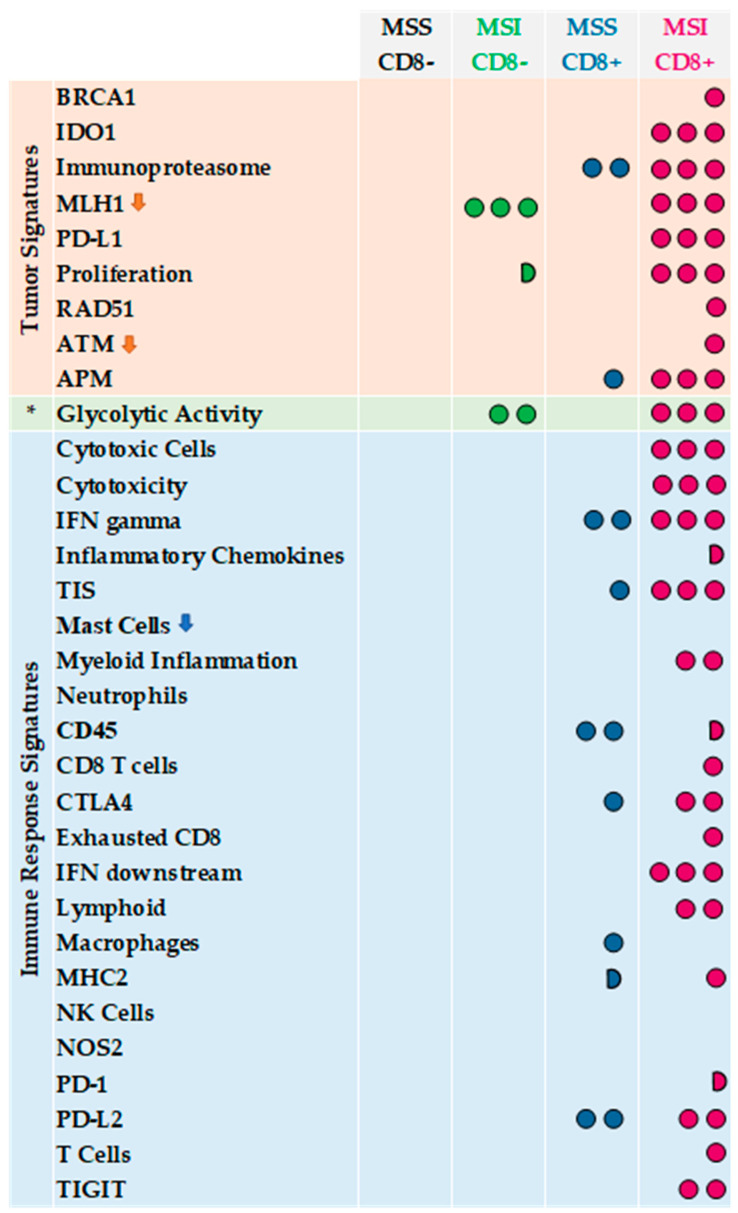
**Tumor**, **tumor microeviroment** (*) and **immune response** signatures that are significantly overexpressed/downregulated in **MSI/CD8+**, **MSS/CD8+** and **MSI/CD8+** groups versus **MSS/CD8−** (reference group). Three dots are for *p* < 0.0001, two dots are for *p* = 0.0001–0.01; one dot is for *p* = 0.02–0.05; half dot is for *p* = 0.06–0.07; the arrow indicates a downregulation of the signature.

Finally, in Figure 3 we summarized GEPs considering the following four tumor categories: 27 MSI/CD8+, 34 MSS/CD8− (reference group), 12 MSS/CD8+ and 15 MSI/CD8− CRCs. From this schematic representation, MSI/CD8+ showed the overexpression/downregulation of 27 signatures with respect to MSS/CD8− cases. Interestingly, MSS/CD8+ CRCs were characterized by an upregulation of seven immune signatures (TIS, IFN gamma signaling, MHC2, CD45, CTLA4, and PD-L2) and overexpression of tumor-intrinsic responses such as APM and the signature measuring key components of the immunoproteasome. By contrast, MSI/CD8- CRCs were very similar to MSS/CD8- cases, with the only exception for the downregulation of MLH1 and for the upregulation of glycolytic activity and tumor proliferation.

### 3.4. Survival Analysis

**Figure 4:**
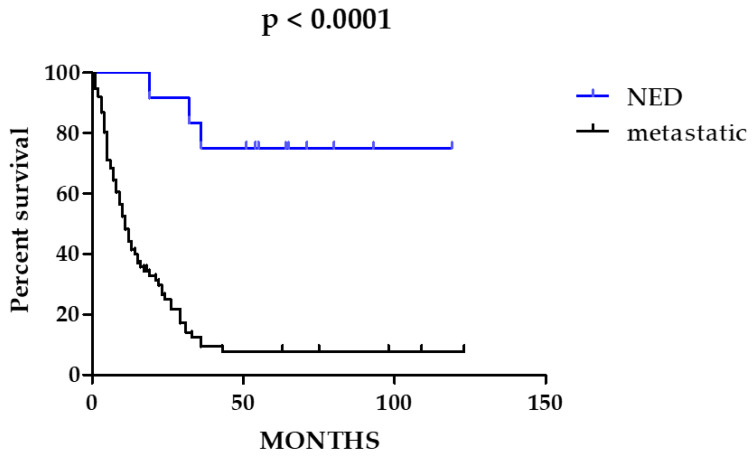
Overall Survival (OS) analysis of metastatic versus non-metastatic CRC patients. In blue, the survival curve of non-metastatic patients (no evidence of disease, NED). In black, the survival curve of metastatic patients.

Overall Survival analysis (OS) was performed for all 88 patients comparing the OS of the 76 patients affected by stage IV CRCs with OS of the 12 patients with no evidence of metastatic disease, NED), as the 12 NED patients showed a significantly better prognosis (Figure 4, *p* < 0.0001), they were excluded from further survival analyses.

Considering the subset of metastatic patients (n = 76) exclusively, none of the analyzed signatures resulted significantly associated with prognosis (Table S5). To note, none of the patients included was treated with immunotherapy because in Italy, it was not available for the treatment of mCRC MSI-high at the time of the enrollment in this study.

### 3.5. Tumor Inflammation Signature (TIS) within BRAF-CRCs and Correlation with CD8+ TIL Content and with MSI

TIS is a weighted metagene signature originally described as a biomarker predictive of response patients with different cancer types, treated with pembrolizumab in the context of clinical trials [21].

**Figure 5:**
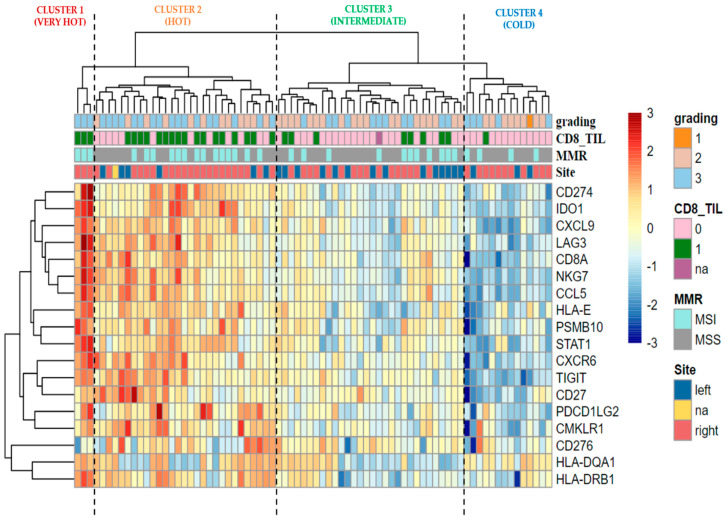
The ‘TIS’ heatmap uses unsupervised hierarchical clustering to show relatedness among the 18 genes in TIS for each sample. Z-scores are calculated based on each gene to have a mean of zero and a standard deviation of one. The standardized signature scores are truncated at ±3 standard deviations to preserve greater clarity in color change within the largest proportion of data (99% of the data should fall within ±3 standard deviations of the mean). Sample annotations are listed at the top of the heatmap: grading; CD8 positive (category 1) vs. CD8 negative (category 0) CRCs (pink vs. green); MSI vs. MSS tumors (aquamarine vs. grey); anatomical site of onset left vs. right (blue vs. red). Na, not available data; MMR, mismatch repair status; MSI, microsatellite unstable; MSS, microsatellite stable.

We performed an unsupervised hierarchical clustering analysis of TIS signature only considering the 76 metastatic BRAF-CRCs, and we identified four major clusters (Figure 5): cluster 1 characterized by a very hot signature profile (3 BRAF-CRCs, 3.9%), cluster 2 with a hot profile (29 BRAF-CRCs, 38.2%), cluster 3 with an intermediate profile (30 BRAF-CRCs, 39.5%), and cluster 4 showing a cold profile with the downregulation of almost all TIS genes (14 BRAF-CRCs, 18.4%). Twenty out of 29 (69%) CD8+ BRAF-CRCs are enlisted in cluster 1 or 2, showing a hot signature profile. The remaining nine CD8+ cases fell in intermediate cluster 3 (eight cases, 24%) or in cluster 4 (1 case, 3%). As regards the correlation of TIS clusters with MSI status, BRAF-CRCs with MSI were distributed as follows: 17 cases in cluster 1 or 2 (53.1%), 11 CRCs in cluster 3 (34.4%), and four tumors in cluster 4 (12.5%).

**Figure 6:**
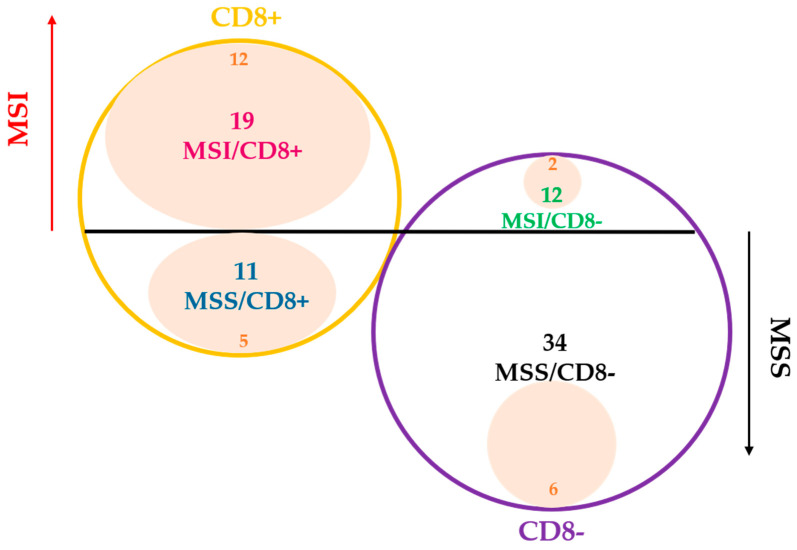
Graphic distribution of TIS high CRCs (pink areas, TIS > 7.274) among metastatic CRCs (n = 76) considering both MSI status (MSI red arrow vs. MSS black arrow) and CD8 TIL content (CD8+ yellow circle vs. CD8− purple circle).

Notably, a high TIS score (upper tertile, TIS > 7.274) was significantly associated to a CD8+ TIL content (68% CD8+ vs. 32% CD8-, *p* = 0.0005) but it was not correlated with MSI (56% MSI vs. 44% MSS, NS) (Figure 6).

## 4. Discussion

BRAF-mCRCs are considered a unique clinical, morphological, therapeutic entity characterized by a dismal prognosis and that do not respond efficiently to both standard chemotherapy and to orally selective inhibitors of BRAF^V600E^. Moreover, despite the well-known association between BRAF mutations and the presence of MSI in sporadic CRCs (40–60% of CRCs and 3–5% of mCRCs), it is not clear whether tumor-intrinsic factors and the microenvironment are potential targets for ICIs in at least a subset of these patients.

For the first time, in this study, GEPs were used to analyze immune-related pathways in BRAF-CRCs, identifying a “hot/inflamed profile” in a high fraction of these tumors (52% of cases; Cluster 1 in Figure 1). Moreover, considering TIS signature in only BRAF-mCRCs, we found 32 (42%) BRAF-mCRCs exhibiting a hot signature profile (cluster 1 and cluster 2 in Figure 5). TIS signature was recently proposed as a pan-cancer measurement of the inflamed tumor phenotype and was associated with objective response to pembrolizumab across a variety of tumors [22]. Our results suggested that a significant fraction of BRAF-mCRCs may be a candidate for ICIs, some of which are classified as MSS-CRCs and would not be selected for ICIs therapy [21]. In fact, the present study found only a partial overlap between these hot signatures and MSI, leading to two considerations: firstly, a subset of MSI tumors showed a “cold” profile (42% in Figure 1 and 47% in Figure 5). This might explain why only 70% of MSI-CRCs are sensitive to ICIs [4,22], confirming that MSI is only a surrogate measure of the intrinsic potential tumor immunogenicity, being upstream of the immune response cascade. Secondly, a not negligible proportion of MSS cases might be sensitive to ICIs. In line with these considerations, we found that APM signature was not significantly upregulated in MSI BRAF-CRCs compared with MSS cases (Figure 2), demonstrating that the abundance of genes in the MHC Class I antigen presentation pathway and key genes involved in processing the antigens prior to presentation, were not differentially expressed in the two subsets of tumors. This finding is in line with many previous works that described the absence of HLA-I in both MSI and MSS CRCs [23,24,25,26,27,28,29]. This is a crucial point because HLA class I loss results in tumor immune escape from cytotoxic T lymphocytes during the natural history of CRC development [30]. In this context, there is increasing evidence indicating that immunotherapy is effective in eliminating HLA-I positive tumor cells, while cells with loss or downregulation of HLA-I escape the therapy-induced immune response and produce new distant tumor lesions [31]. The recovery of HLA-I antigens is a major future challenge in predicting resistance to ICIs. Thus, it will be particularly important to recognize the main molecular mechanisms underlying the HLA-I alterations in cancer cells. Some authors recently suggested that the progression or regression of a tumor lesion in cancer patients undergoing immunotherapy could be predetermined by the molecular mechanism responsible for the MHC Class I alteration. Therefore, HLA-I downregulation produced by reversible molecular alterations can be corrected in vitro by IFN-γ and other cytokines [32,33] or recovered by immunotherapy that can stimulate a release of T-helper type I (TH1) cytokines in the tumor microenvironment [34]. By contrast, immunomodulator factors cannot correct irreversible alterations caused by mutational events and chromosomal abnormalities in HLA-I and Beta-2-Microglobulin (β*2m*) genes and the IFN signaling pathway, leading to the progression of HLA-I negative lesions [31,35,36].

HLA-I loss has been seen in up to 70% of MSI CRCs and is primarily due to a lack of β2m synthesis or to the synthesis of a truncated β2m caused by mutations or loss of heterozygosity [37,38,39,40,41].

By contrast, in the present work, APM signature as well as MHC class II antigen presentation were significantly upregulated in CD8+ BRAF-CRCs compared with CD8− tumors. In addition, many other signatures were overexpressed in just CD8+ BRAF-CRCs and not in MSI BRAF-CRCs (Figure 2), including immune response profiles measuring lymphoid, macrophage, NK, CD45, and CD8 T cells abundance, interferon signaling response and Nitric Oxide Synthase 2 gene expression, exhausted CD8 cells and inhibitory tumor mechanisms that suppress anti-tumor immune activity including upregulation of PD-L2, PD-1, TIGIT, CTLA4.

Thus, in this work, GEPs demonstrated that despite the positive correlation between MSI and CD8+ TIL content (Table 1, *p* = 0.0001), the two subsets of tumors show different immune profiles with only a partial overlap of differential expressed signatures (Figure 2). Our results highlighted that the evaluation of CD8+ TIL content in CRCs is a direct measure of the ongoing immune response within the tumor. In line with these results, Chalabi M et al. [5] showed that neoadjuvant immunotherapy leads to pathological responses in MMR-proficient and MMR-deficient early-stage CRCs, suggesting that CD8+PD-1+Tcell infiltration was the sole biomarker found to predict response in MMR-proficient CRCs. Thus, this work confirmed our preliminary results obtained with a smaller series of BRAF-mCRCs [17], in which we suggested that the combination of the two biomarkers (MSI status and CD8+ TIL content) could increase diagnostic accuracy in identifying patients who can potentially benefit from ICIs. Accordingly, MSI/CD8+ and MSS/CD8- immune profiles reported in Figure 3 appear to be two mirror images since 27 signatures are overexpressed/downregulated in MSI/CD8+ in comparison to MSS/CD8− cases.

As expected, MSI/CD8+ tumors were characterized by a wide upregulation of both tumor-intrinsic factors involved in tumor immunogenicity/tumor escape from immune surveillance and immune response, including inhibitory immune signaling and anti-tumor immune activity.

Interestingly, while MSS/CD8+ CRCs were characterized by an upregulation of seven immune signatures (TIS, IFN gamma signaling, MHC2, CD45, CTLA4, and PD-L2) and the overexpression of tumor-intrinsic responses such as APM and the signature measuring key components of the immunoproteasome, MSI/CD8− tumors did not show any of them. This result suggests that a fraction of MSS BRAF-CRCs have potential as a target for immune checkpoint inhibitors.

This study has some limitations. The tumors included in this series were from a retrospective cohort of immunotherapy-naïve BRAF-CRCs selected from routine diagnostics (in Italy, immunotherapy was not available at the time of the enrollment in this study), and we could study only the primary tumors of the samples without comparison with the matched metastases of the patients. Moreover, it is necessary to obtain clinical validation of these results in patients with BRAF mutant mCRC receiving ICIs.

Another limitation of the current study is that it relies only upon CRCs with BRAF mutation, which represent a minor fraction of all colorectal carcinomas. Thus, the conclusions of the present analysis deserve to be deeply investigated in all CRCs, regardless of BRAF mutational status. Despite these limitations, the present work is the first report to show immune-related profiles of a large and well-characterized cohort of BRAF-CRCs selected from routine diagnostics, producing robust data from archival samples of FFPE tissues and correlating the molecular data with a wide panel of clinical-pathological features available for all tumors.

Our study demonstrates that GEPs, integrating both tumor intrinsic factors and pre-existing adaptive immune response, is a promising strategy to improve the knowledge of the mechanisms involved in the tumor resistance to immune checkpoint blockade in CRC and to expand the spectrum of patients who can benefit from immune checkpoint blockade.

## 5. Conclusions

A significant fraction of BRAF-mCRCs shows a hot profile/high TIS scores and, for this reason, may be potential candidates for responding to ICIs.

Only a partial overlap between these hot signatures and MSI was observed, demonstrating that MSI should not be the unique marker to select patients for ICIs therapy and that a not negligible proportion of MSS cases, which are CD8+, might benefit from immunotherapy.

A combination of MSI status and CD8+ TIL content could therefore increase accuracy in identifying patients who can potentially benefit from ICIs.

The analysis of GEPs is a promising strategy both for immune profiling of primary tumors before any treatment and for following the evolution of metastatic disease during therapy.

## Figures and Tables

**Table 1:** Clinico-pathological characteristics of BRAF-CRCs based on MMR status and on CD8+ TIL infiltrate.

Variable	All Cases ^§^ (n = 89)	MSI CRC (n = 43)	MSS CRC (n = 46)	*p* Value	CD8+ CRC ^#^ (n = 39)	CD8- CRC ^#^ (n = 49)	*p* Value
**MMR (MSI)**	43 (48%)	-	-	-	**27 (69%)**	15 (31%)	0.0001
**CD8+ (n = 88)**	39 (44%)	**27 (64%)**	12 (26%)	**0.0001**	-	-	-
**Age, y (mean ± SD)**	69.8 ± 10.1	**72.1 ± 8.6**	67.7 ± 11.1	**0.0311**	72.1 ± 8.8	68.1 ± 11	ns (0.07)
**Female sex**	**54 (61%)**	28 (65%)	26 (57%)	ns	22 (56%)	31 (63%)	ns
**Right tumor site**	**64 (73%)**	**39 (91%)**	25 (56%)	**<0.0001**	31 (79%)	32 (67%)	ns
**Histologic type**				**0.004**			**0.005**
Tubular	38 (43%)	12 (28%)	**26 (57%)**		10 (26%)	**27 (55%)**	
Mucinous/signet ring cells	33 (37%)	**21 (49%)**	12 (25%)		**20 (51%)**	13 (27%)	
Medullary/undifferentiated	12 (13%)	9 (21%)	3 (7%)		8 (21%)	4 (8%)	
MANEC	6 (7%)	1 (2%)	**5 (11%)**		1 (3%)	**5 (10%)**	
**High Grade**	43 (48%)	**27 (63%)**	16 (35%)	**0.008**	23 (59%)	19 (39%)	ns (0.08)
**Stage at diagnosis (I-III)**	44 (49%)	**27 (63%)**	17 (37%)	**0.015**	22 (56%)	21 (43%)	ns
**Distant Metastases (n = 79)**				**0.0013**			**0.0291**
Presence *	77 (86,5%)	32 (74%)	45 (98%)		30 (77%)	46 (94%)	
Absence	12 (13,5%)	**11 (26%)**	1 (2%)		9 (23%)	3 (6%)	
**Infiltrative Growth pattern (n = 83)**	64 (77%)	30 (70%)	40 (87%)	ns	27 (69%)	42 (86%)	ns
**Stroma ≥ 20% (n = 87)**	52 (60%)	18 (43%)	**34 (76%)**	**0.0023**	13 (34%)	21 (44%)	ns
**Synaptophysin ≥ 1 (n = 77)**	16 (21%)	1 (3%)	**15 (39%)**	**0.0001**	1 (3%)	**15 (33%)**	**0.001**
**p53 ≥ 50 (n = 67)**	22 (34%)	2 (7%)	**20 (51%)**	**0.0002**	3 (14%)	**19 (43%)**	**0.0255**
**PD-L1 ≥ 1 (n = 65)**	17 (26%)	**16 (94%)**	1 (6%)	**<0.0001**	**14 (82%)**	3 (18%)	**<0.0001**

Legend: ^§^ one patient had two distinct BRAF-CRCs, one was MSI (ID 54), and one was MSS (ID 75; ^#^ CD8 analysis was possible on 88 out of 89 cases; ns = not significant; * 40 patients showed multiple sites of metastasis. CRC = colorectal cancer; MMR = DNA mismatch repair; MSI = Microsatellite instability; MSS = Microsatellite Stable; CD8 + = cytotoxic T lymphocytes; MANEC = Mixed adeno-neuroendocrine carcinoma; PD-L1 = Programmed Death-Ligand 1. The bold is for significant *p*-values.

**Table 2:** PanCancer IO 360 Biological Signatures.

Tumor Signature			Tumor Microenvironment Signature		Immune Response Signature		
Tumor Immunogenicity	Tumor Sensitivity to Immune Attack	Inhibitory Tumor Mechanisms	Stromal Factors	Inhibitory Metabolism	Inhibitory Immune Signaling	Anti-TumorImmune Activity	Immune Cell Population Abundance
APM	Apoptosis	B7-H3	Endothelial Cells	Glycol Act	ARG1	TIS	B Cells
APM Loss	JAK-STAT Loss	IDO1	Stroma	Hypoxia	CTLA4	Cytotoxicity	CD45
Hypermutation	Proliferation	PD-L1			IL10	IFN Down	CD8 T Cells
Immunoproteasome		TGF-Beta			Inflam Chemokine	IFN Gamma	Cytotoxic Cells
MAGEs					Myeloid Inflam	Lymphoid	DC
MMR Loss					NOS2	MHC2	Exhausted CD8
MSI Predictor					PD-1	Myeloid	Macrophages
					PD-L2		Mast Cells
					TIGIT		Neutrophils
							NK CD56dim
							NK Cells
							T Cells
							TH1 Cells
							Treg

Legend: APM = Antigen presenting (or processing) machinery; MAGEs = Melanoma-Associated Antigen Gene Expression; MMR Loss = Mismatch Repair Loss; MSI = Microsatellite Instability; JAK-STAT Loss = JAK-STAT pathway expression loss; B7-H3 = CD276 gene expression; IDO1 = Indoleamine 2,3-dioxygenase 1 gene expression; PD-L1 = Program cell death ligand 1 gene expression; TGF-Beta = Transforming Growth Factor Beta gene expression; ARG1 = Arginase-1 gene expression; CTLA4 = Cytotoxic T-lymphocyte-associated protein 4 gene expression; IL10 = Interleukin-10 gene expression; NOS2 = Nitric Oxide Synthase 2 gene expression; PD-1 = Program cell death receptor 1 gene expression; PD-L2 = Program cell death ligand 2 gene expression; TIGIT = T cell immunoreceptor and Ig and ITIMS gene expression; TIS = Tumor Inflammation Signature, TIS measures the abundance of a peripherally suppressed adaptive immune response within the tumor; IFN Down = Interferon Signaling Response; IFN Gamma = Interferon gamma signaling; MHC2 = Major histocompatibility complex class II antigen presentation; DC = Dendritic cell abundance; NK = Natural Killer; TH1 Cells = T-box transcription factor TBX21 (T-bet) expressing cell abundance; Treg = Regulatory T cell abundance.

## Data Availability

The data presented in this study are available in the article or in supplementary material.

## Supplementary Materials

The following are available online at https://www.mdpi.com/article/10.3390/cancers14163951/s1, Table S1: Comprehensive clinical, histological, immunohistochemical, and molecular features of 89 BRAF-CRCs, Table S2: Immunohistochemistry primary antibody list, Table S3: “All Signatures” and “All Genes” results tables used to generate the Figure 2a,b, Table S4: “All Signatures” and “All Genes” results tables used to generate the Figure 2c,d, Table S5: Overall survival results.
